# *Blastocystis* and *Giardia duodenalis* infection in a male prison in Spain

**DOI:** 10.1016/j.parepi.2024.e00407

**Published:** 2024-12-30

**Authors:** Carla Muñoz-Antoli, Jacklyn Comas, María José Irisarri-Gutiérrez, Lucrecia Acosta, José Guillermo Esteban, Rafael Toledo

**Affiliations:** a Department of Pharmacy and Pharmaceutical Technology and Parasitology, Faculty of Pharmacy, University of Valencia, Valencia, Spain; b Grupo de Investigación Salud y Comunidad, Línea de Enfermedades Infecciosas Tropicales, Universidad Tecnológica del Chocó Diego Luis Córdoba, Chocó, Colombia; c Department of Preventive Medicine, Public Health and Microbiology, Faculty of Medicine, Autonomous University of Madrid, Madrid, Spain; d Parasitology Area, Department of Agrochemistry and Environment, Miguel Hernández de Elche University, Alicante, Spain

**Keywords:** *Blastocystis*, *Giardia duodenalis*, Human infection, Prison, Inmates, Spain

## Abstract

**Background:**

General conditions in a prison may facilitate water- or food-borne infections.

**Methods:**

Detection of intestinal parasites was achieved in 471 male prison inmates by standard microscopic procedures on their stool samples. Positive samples were processed by PCR amplification of a 600-bp fragment of the *Blastocystis* SSU rRNA gene and partial sequences of the *Giardia duodenalis bg* genes. Identification of subtypes/genotypes was based on Sanger sequencing methods.

**Results:**

*Blastocystis* was found in 7.9 % (37/471) and *G. duodenalis* was found in 2.1 % (10/471). Out of the 37 *Blastocystis* positive samples, 54 % (20/37) were successfully subtyped, allowing the identification of the subtypes ST3 (50 %), ST1 (25 %), ST2 (15 %), ST4 (5 %) and ST6 (5 %). Out of 10 *G. duodenalis* positive samples, 50 % (5/10) were successfully genotyped, allowing the identification of genotypes A (80 %) and B (20 %).

**Conclusions:**

The predominance of ST3 within the prison inmates, together with its low intra-ST genetic variability, reflected inter-human transmission with spatial stability. The *G. duodenalis* distribution is not wide enough to consider the possibility of a generalized transmission via contaminated water or food. Personal hygiene practices among male prison inmates may be an important measure to prevent the transmission.

## 1 Introduction

In closed institutions such as penitentiary centers, epidemic contamination/infections can affect a large number of inmates. Commonly, water- or food-borne infections are the most frequent. Intestinal parasites are often transmitted using these routes and conditions in a prison may facilitate their transmission. Muñoz-Antoli et al. (Muñoz-Antoli et al., 2023) just studied the prevalence of intestinal parasites in the Centro Penitenciario Picassent (CPP) (Valencia, Spain). Among the parasites detected in that work, the presence of *Blastocystis* and *G. duodenalis* was the most relevant.

*Blastocystis* is included in the “Water Sanitation and Health Program” of the World Health Organization, and in the list of waterborne parasites (WHO, 2011), although uncertainty about its pathogenic character persists even today. Several common intestinal symptoms have been attributed to *Blastocystis* infection (diarrhoea, abdominal pain, irritable bowel syndrome and cutaneous lesions) (Khorshidvand et al., 2021; El Safadi et al., 2016). In addition, the pathogenicity of certain subtypes has also been proven in diarrheal patients, such as the case of *Blastocystis* ST7, which produces a dysbiosis microbiota, with a lower bacterial diversity and altered microbial structure (Deng et al., 2022a; Deng et al., 2022b). In contrast, other studies have not found a link between *Blastocystis* and disease (Leder et al., 2005; Ozyurt et al., 2008). Recently, *Blastocystis* has also been related to healthy gut microbiome and a lower incidence of inflammatory diseases (Deng et al., 2021; Matovelle et al., 2022). It should be commented that, at an experimental level in different murine colonization models, it has already been demonstrated how *Blastocystis* ST4 exerts beneficial effects on intestinal commensal bacteria (Deng and Tan, 2022) and even producing an amelioration of colonic inflammation, likely through immunomodulatory effects of short-chain fatty acids (SCFAs), Th2 and T reg effectors (Deng et al., 2022c). Similarly, *Blastocystis* ST1 colonization increased the proportion of beneficial bacteria and induced Th2 and Treg cell responses in normal healthy mice (Deng et al., 2023). As *Blastocystis* displays a high degree of genetic diversity, it has been suggested that knowing the subtype of *Blastocystis* may be essential to explain the pathology caused by the parasite (Stensvold et al., 2011; Domínguez-Márquez et al., 2009) and also essential to determine the possible routes of infection in a community.

*G. duodenalis* is regarded as a relevant diarrhoea-causing pathogen transmitted either through contact with infected humans or animals or via ingestion of contaminated food or water. *G. duodenalis* exhibits a certain degree of genetic diversity, and a total of eight assemblages (A to H) can be differentiated within the complex. Human infections are caused mainly by assemblages A and B and, to a lesser extent, by other assemblages, comprising assemblage C and D (dogs), assemblage E (domestic and wild ungulates), assemblage F (cats), assemblage G (mice and rats) and assemblage H (marine mammals) (Ryan et al., 2021; Asghari et al., 2023).

Generally, prison inmates consider that they receive poor quality food and water, which could be the source of their infections. In the present paper, we analyze the genetic diversity of *Blastocystis* and *G. duodenalis* in the inmates of CPP, going a little deeper into our previous work, in order to determine the potential routes of transmission of these intestinal pathogens in an environment with these characteristics, and to know if the infection is related to a water or food contamination.

## 2 Methods

### 2.1 Study population and design

The Centro Penitenciario Picassent (CPP, Valencia, Spain) (39°35′N, 0°45′O coordinates) is one of the main prisons of Spain, housing approximately 2100 inmates. The CPP was chosen due to its accessibility from the University of Valencia (convenient for sample transportation) and high population of male inmates. From April–June 2022, a cross-sectional survey, compulsory for all prison inmates of 11 male's CPP prison modules, was carried out. We obtained a unique fresh faecal samples from 471 male inmates. Together with the stool sample (about 8 g in a plastic bottle with screw cap) an anonymous questionnaire was obtained for every participant inmate, including demographic data (age, sex, nationality), intestinal symptomatology and prison characteristics (time, exit permit).

### 2.2 Microscopic examination

**Fig. 1:**
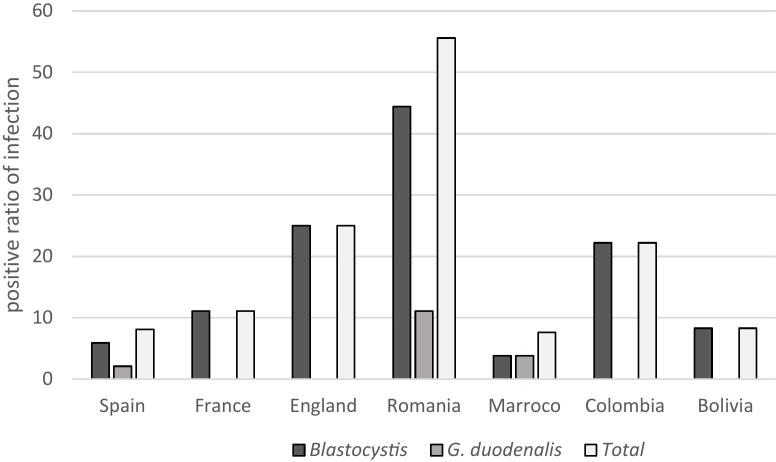
The total infection ratio in each country: Blastocystis and G. duodenalis ratio in the different inmates´ nationalities

*Blastocystis* and/or *G. duodenalis* were identified with high sensitivity thanks to our previous microscopic experience with faecal samples (Muñoz-Antoli et al., 2023) (Suplemental Fig. 1 a,b). *Blastocystis* positivity was considered when an intensity of at least 5 cyst/vacuolar/trophozoite forms was reached (Speich et al., 2013). The detection of just one *G. duodenalis* cyst indicated positivity.

### 2.3 DNA extraction

DNA was extracted from *Blastocystis* and/or *G. duodenalis* microscopy-positive faecal samples to increase specificity characterizing the *Blastocystis* subtypes and *G. duodenalis* assemblages. Aliquots of 200 mg of frozen faecal material were weighed into sterile microcentrifuge tubes. Genomic DNA extraction was performed using a QIAamp DNA Stool Mini Kit (QIAGEN) according to the manufacturer's instructions. DNA was eluted in 200 μL buffer AE, purified in molecular grade water (200 μL) and stored at −20 °C.

### 2.4 PCR and sequencing

The method described by Scicluna et al. (Scicluna et al., 2006) was used for *Blastocystis* subtype analysis. Direct PCR amplification of *Blastocystis* was performed, using the barcoding region primers BhRDr (5′–3′: GAGCTTTTTAACTGCAACAACG) and RD5 (5′–3′: ATCTGGTTGATCCTGCCAGT), targeting a 600-bp fragment of the small subunit ribosomal RNA gene (SSU rRNA). The 25 μL reaction mixture included: 5 μL template DNA; 0.5 μM of each primer; 3.5 mM MgCl2; 200 μM dNTPs: 1 U Taq DNA polymerase (Thermo Scientific); and, 1× Taq reaction buffer. PCR conditions consisted of 35 cycles of initial denaturation at 95 °C for 2 min, followed by denaturation at 94 °C for 30 s, annealing at 60 °C for 30 s, extension at 72 °C for 30 s, and final extension at 72 °C for 1 min, carried out in a C1000 MJ mini-thermal cycler.

In the case of *G. duodenalis* a multilocus sequence typing scheme based on the amplification of partial sequences of β-giardin (*bg*) genes was used for genotyping purposes (Seguí et al., 2018; Hernández-Castro et al., 2023). A nested-PCR protocol was used to amplify a ∼ 511-bp fragment of the *bg* gene of *G. duodenalis*. PCR reactions were conducted in a final volume of 25 μL consisting of 3 μL of genomic DNA and 0.4 μM of the primers pairs G7_F/G759_R in the primary reaction and G99_F/G609_R in the secondary reaction. Cycling parameters for the primary PCR reaction were an initial step of 95 °C for 7 min, followed by 35 cycles of 95 °C for 30 s, 65 °C for 30 s, and 72 °C for 1 min with a final extension of 72 °C for 7 min. The same conditions were used in the secondary PCR except that the annealing temperature was 55 °C (Seguí et al., 2018).

All PCR experiments contained a negative control (4 μL of nuclease-free water) for contamination detection. The PCR products were electrophoresed in 2 % agarose gel stained with Safe View Nucleic Acid Stain (NBS Biologicals Ltd., England) along with a 100 bp Plus DNA Ladder (Fermentas, Life Sciences) as a standard size.

The purified samples, with their respective primers, were sent to the Central Service for Experimental Research Support (SCSIE), where they performed the sequencing by capillary electrophoresis with BigDye® Terminator Chemistry (Applied Biosystems in both forward and reverse directions (F and R) using the primers described for the PCRs and an automated sequencer ABI PRISM 3130.

Subtypes were determined using the sequence query facility in the *Blastocystis* SequenceTyping website available at http://pubmlst.org/blastocystis/ (Stensvold et al., 2007).

In the case of *G. duodenalis*, raw sequencing data in both forward and reverse directions were viewed using the Chromas Lite version 2.1 sequence analysis program. Generated DNA consensus sequences were aligned to appropriate reference sequences using MEGA version 6 software to identify *Giardia* assemblages/subassemblages (Tamura et al., 2013).

## 3 Results

### 3.1 Demographic and microscope results

The infection appeared in 9.9 % (47/471) of CPP male inmates with a mean of 42 years of age (range 23–62) and among those who spent a mean of 2 years in prison (range 1–11) (Table 1). Being a Spanish inmate implies a statistical risk to present infection (*p* = 0.012), reaching the 63.8 % (30/47) of infections. Only 14.8 % (7/47) of the infected inmates referred to a recent exit prison permit. Among those with *Blastocystis* 18.9 % (7/37) presented diarrhoeal stool samples and 8.1 % (3/37) abdominal pain. None of those with *G. duodenalis* were symptomatic. No other intestinal symptomatology was referred among those infected.

**Table 1:** Demographic and microscope infection results obtained in the 471 CPP male inmates analyzed. Bivariate analysis of risk factors (OR 95 %CI = Odds ratio 95 % confidence interval).

		*Blastocystis*		*G. duodenalis*		Total Infected			
*N* = 471		*n* = 37	7.9 %	*n* = 10	2.1 %	n = 47	9.9 %		
Years of age	mean	40		45		42			
	min	23		30		23			
	max	60		62		62			
Years in prison	mean	2		2		2			
	min	1		1		1			
	max	11		9		11		OR (95 %CI)	*p*-value
Nacionality	Spanish	22	59.5 %	8	80 %	30	63.8 %	0.42(0.22–0.80)	0.012
	others	15	40.5 %	2	20 %	17	36.1 %		
Exit permit	yes	4	10.8 %	3	30 %	7	14.8 %	2.00(0.83–4.82)	0.189
	no	33	89.2 %	7	70 %	40	85.1 %		
Diarrhoea	yes	7	18.9 %	0	–	7	14.8 %	1.63(0.68–3.88)	0.384
	no	30	81.0 %	10	100 %	40	85.1 %		
Abdominal pain	yes	3	8.1 %	0	–	3	6.4 %	2.82(0.74–10.64)	0.259
	no	34	91.9 %	10	100 %	44	93.6 %		

The prevalence of *Blastocystis* infection in prison inmates was 7.9 % (37/471) and that of *G. duodenalis* was 2.1 % (10/471) identified by microscopic examination. No mixed infections were observed (Table 1). *Blastocystis* appeared in seven different inmates´ nationalities of whom 59.5 % (22/37) were Spanish. *G. duodenalis* appeared in three different inmates´ nationalities, with being Spanish the most infected (80 %) (8/10). The infection ratio in each country shows that Romanians (55.6 %) are the most frequently infected (*p* < 0.0001). Spanish, Romanians and Moroccans inmates presented both *Blastocystis* and *G. duodenalis* infection (Fig. 1).

**Fig. 2:**
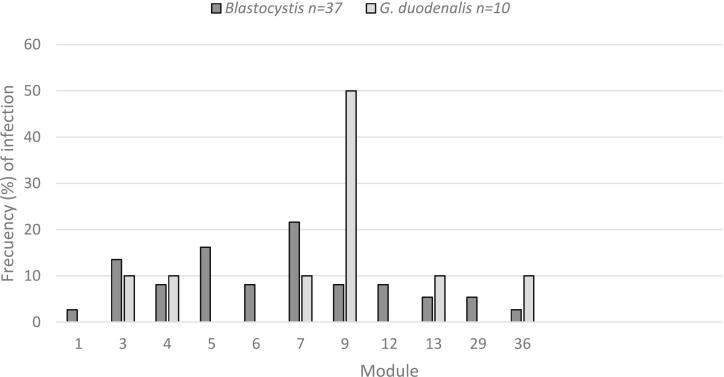
Distribution of *Blastocystis* and *G. duodenalis* infection according to the CPP prison modules

The distribution of *Blastocystis* and *G. duodenalis* infection according to the CPP prison modules is shown in Fig. 2. *Blastocystis* infection was the most widely distributed affecting all the modules equally, without significant differences (*p* = 0.374). Similarly, *G. duodenalis* infection reached the highest values in just one module but without significant differences (*p* = 0.181) between modules. The infection of both parasites concurred in 6 modules.

### 3.2 Molecular results

Despite the accuracy of microscopic examinations, just 54 % (20/37) of the *Blastocystis*-positive human samples were successfully sequenced. The identified *Blastocystis* subtypes were assigned to ST1 (25 %, 5/20), ST2 (15 %, 3/20), ST3 (50 %, 10/20), ST4 (5 %, 1/20) and ST6 (5 %, 1/20) (Supplemental Fig. 2). No mixed subtype infections were identified. Among the symptomatic prison inmates, *Blastocystis* ST3 appeared in 28.6 % (2/7) of those with diarrhoea and in 66.6 % (2/3) of those with abdominal pain.

**Fig. 3:**
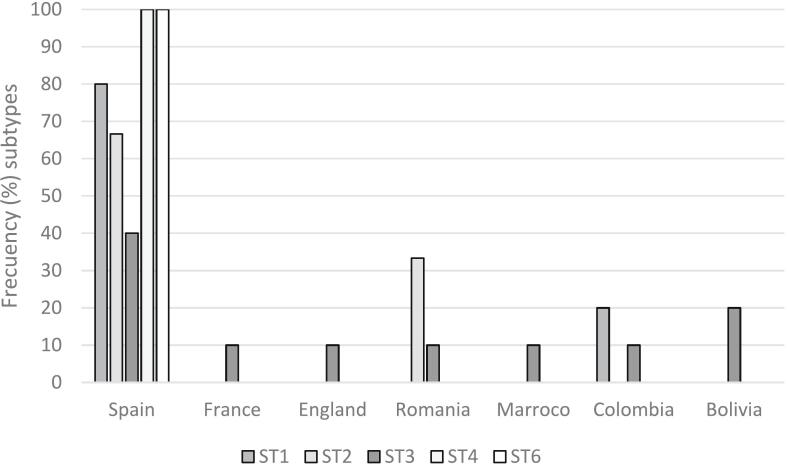
Distribution of different Blastocystis subtypes detected according to the inmates´ nationality

The distribution of different subtypes detected according to the inmates´ nationality is shown in Fig. 3. Only ST3 is the most widely distributed subtype. Furthermore, all different subtypes detected are housed by Spanish inmates.

Allele analysis of the *Blastocystis* 18S rRNA gene for each subtype detected a total of 7 different variants within the samples. ST1 (alleles 2 and 4) and ST2 (alleles 9 and 11) showed higher allelic diversity than ST3 (allele 34, 50 %), ST4 (allele 42, 5 %) and ST6 (allele 123, 5 %). In ST1 and ST2 allele 4 (20 %; 4/20) and allele 9 (10 %; 2/20) were the most frequent, respectively (Supplemental Fig. 3).

All 10 *G. duodenalis* -positive human samples at microscopy examination were subjected to the amplification of partial sequences of the *bg* gene. Only a nested PCR of the *bg* gene was resolved to specify the assemblage involved. Of them, 50 % (5/10) were successfully genotyped at this locus. Assemblage A (80 %, 4/5) and Assemblage B (20 %, 1/5) were detected, and their distribution according to the inmates´ nationality is shown in Supplemental Fig. 4. Although the molecular diversity of *G. duodenalis* at the subassemblage level was investigated using the *bg* gene as a genetic marker no successful sequencing data were generated.

## 4 Discussion

Our study provides the first report on the genetic diversity of *Blastocystis* and *G. duodenalis* in an understudied population, such as Spanish prison inmates, which may provide relevant information to determine how the transmission of intestinal pathogens occurs. In fact, the results obtained suggest that human-to-human is the most relevant route of transmission of intestinal parasites in an environment as particular as a prison.

*Blastocystis* was found to be the most represented parasite (7.9 %), similar to our previous results (7.0 %) about parasites prevalence and related risk factors in the inmate population of CPP (Muñoz-Antoli et al., 2023), and within the range of prevalences (2.5 %–35.5 %) observed in Spain (Matovelle et al., 2022; Hernández-Castro et al., 2023; González-Moreno et al., 2011; *Microorganisms*, 2020; Hidalgo et al., 2019; Martín-Sánchez et al., 1992; Paulos et al., 2018) and in other European countries (6.1 %–24.2 %) (Bart et al., 2013; Lhotská et al., 2020; Masucci et al., 2011). However, this fact should be taken with caution since most of those surveys have been carried out on specific population groups such as children, immigrants or hospitalized people.

Although the main rate of infection involves the Romanian inmates, Spanish inmates show the highest risk of become infected, also observed in a previous work (Muñoz-Antoli et al., 2023), and result the most infected by *Blastocystis* and *G. duodenalis*.

Up to now, 38 subtypes have been described for *Blastocystis*, with ST1 to ST4 being the most common in humans (Domínguez-Márquez et al., 2009; Paulos et al., 2018; Jiménez et al., 2023; Maloney et al., 2023). Our results show *Blastocystis* ST3 as the most prevalent in the inmates of CPP. Moreover, the subtype *Blastocystis* ST6 was also found in a single sample. In a prison in Malaysia (Angal et al., 2015) *Blastocystis* ST3 was also found to be the predominant subtype (75.8 %), although those authors found also other subtypes in their study: ST1 (21.2 %) and ST6 (3.0 %). The wide distribution of ST3 has also been corroborated by several previous works in different countries. In Italy, *Blastocystis* carriers were identified to harbor ST3 ranging from 40 % (Gabrielli et al., 2020) to 46 % (Mattiucci et al., 2016). In Swedish subjects *Blastocystis* ST3 was identified as the most common subtype (47.6 %) in addition to ST4 (20.6 %) (Forsell et al., 2017). In Netherlands patients *Blastocystis* ST3 reached 42 % (Bart et al., 2013). In Egypt, ST3 had the highest prevalence ranging from 45.5 % (Ahmed et al., 2022) to 61.9 % (Souppart et al., 2012). Interestingly, *Blastocystis* ST3 is considered to be the only variant of human origin whose main route of transmission is human-to-human (Nemati et al., 2021).

Allele homogeneity detected in ST3 (allele 34) agrees with the lowest allele diversity observed in Europe (Nemati et al., 2021). Moreover, the predominance of ST3 within the CPP inmates coupled with its low intra-ST genetic variability, reflected a large inter-human transmission together with its spatial stability. Similar results were obtained in patients from Vietnam (Nguyen et al., 2023).

The second most common subtype in our study (ST1) is normally transmitted through the consumption of water contaminated with feces (Darwish et al., 2023; Leelayoova et al., 2008). However, this fact cannot be confirmed because of the limited observed distribution of *G. duodenalis* that shares this transmission route. The distribution of *G. duodenalis* was not homogeneous and did not affect all modules of the prison, which seems to indicate that there is no widespread transmission through contaminated water or food in prison. It should be noted that there are several previous studies that detected a negative association between *Blastocystis* and tap water as a drinking source (Leelayoova et al., 2008).

We have found only two assemblages of *G. duodenalis* (A and B). Although the prevalence of *G. duodenalis* detected among CPP inmates can be considered as low (2.1 %), the genotyping analysis revealed the predominance of assemblage A (80 %) over assemblage B (20 %). This predominance is common in human populations, although the large number of one assemblage or another varies depending on the study (Hernández-Castro et al., 2023; *Microorganisms*, 2020; Köster et al., 2021; Mateo et al., 2014). Strikingly, assemblage B has only been detected among Moroccan prisoners, suggesting, that the most likely infection method must be human-to-human in relation to the close contact that inmates of the same nationality usually maintain.

The present paper has several limitations: only one sample was obtained per participant, which may prevent obtaining results accurately; loss of sensitivity since only samples identified as *Blastocystis* and/or *G. duodenalis* positive by microscopy have been considered for the molecular analysis; poor resolution of the molecular analysis performed, perhaps due to the presence of contamination and/or inhibitors; and, the lack of results about *G. duodenalis* subassemblages prevents deducing in depth the type of transmission involved.

In conclusion, we have studied the occurrence of *Blastocystis* subtypes and *G. duodenalis* assemblages in an environment as particular as a male prison. The higher prevalence of ST3 of *Blastocystis*, together with the two *G. duodenalis* assemblages detected, suggest that human-to-human transmission is the main route of infection with intestinal protozoa among the inmates. Moreover, the low prevalence detected for *G. duodenalis* among CPP inmates is not wide enough to consider the possibility of a generalized infection due to contaminated water or food transmission. Considering that the origin of *Blastocystis* ST3 (the most prevalent and the most widely distributed subtype) is exclusively human, personal hygiene practices among CPP male inmates may be an important measure to prevent the transmission of intestinal pathogens.

## Funding

This work was supported by AICO/2021/236 from Dirección General de Ciencia e Investigación, Consellería de Innovación, Universidades, 10.13039/100011201Ciencia y Sociedad Digital, 10.13039/501100003359Generalitat Valenciana (Valencia, Spain).

## Ethical considerations and informed consent

The study protocol was submitted for approval to the Ethics Committee of Research in Humans of the Ethics Commission in Experimental Research of University of Valencia (ref. no.:1862541). Stool samples were collected after obtaining permission from the Central Government and CPP administration staff. All information and data collected during this study were collected in strict compliance with ethical rules. To avoid power dynamics that could influence an individual's decision, participation was mandatory for all prison inmates in the randomly chosen modules. Informed consent was obtained from all inmates, meaning that the participants were aware of the study's purpose, risks, and benefits.

## Authorship statement

C.M-A. participated in the conceptualization, funding acquisition, formal analysis, investigation, methodology, writing the original draft and review and editing it; J.C. participated in the formal analysis; M.J.I-G. and L.A. participated in the investigation and methodology; J.G.E. participated in the supervision and validation; R.T. participated in supervision, validation, review and editing. All the authors have read and approved the final manuscript.

## CRediT authorship contribution statement

**Carla Muñoz-Antoli:** Writing – review & editing, Writing – original draft, Methodology, Investigation, Funding acquisition, Formal analysis, Conceptualization. **Jacklyn Comas:** Formal analysis. **María José Irisarri-Gutiérrez:** Methodology, Investigation. **Lucrecia Acosta:** Methodology, Investigation. **José Guillermo Esteban:** Validation, Supervision. **Rafael Toledo:** Writing – review & editing, Validation, Supervision.

## Declaration of competing interest

None.

## Appendix A Supplementary data

Supplementary material 1: Microscopic images of Blastocystis (A) and Giardia duodenalis cysts (B) (40X magnification) Image 1

Supplementary material 2: Rate of different Blastocystis subtypes (ST) found in Blastocystis-positive human samples sequenced Image 2

Supplementary material 3: Allele analysis (%) of each Blastocystis subtype detected Image 3

Supplementary material 4: Distribution of G. duodenalis assemblage A and B according to the inmate´s nationalityFootnoteView Edit Log13 Image 4
