# Supplementary figures and images for: Blastocystis and Giardia duodenalis infection in a male prison in Spain
Source: Parasite Epidemiol Control. 2024 Dec 30;28:e00407. doi: 10.1016/j.parepi.2024.e00407 (PMC11780165; doi:10.1016/j.parepi.2024.e00407)

## Slide 1
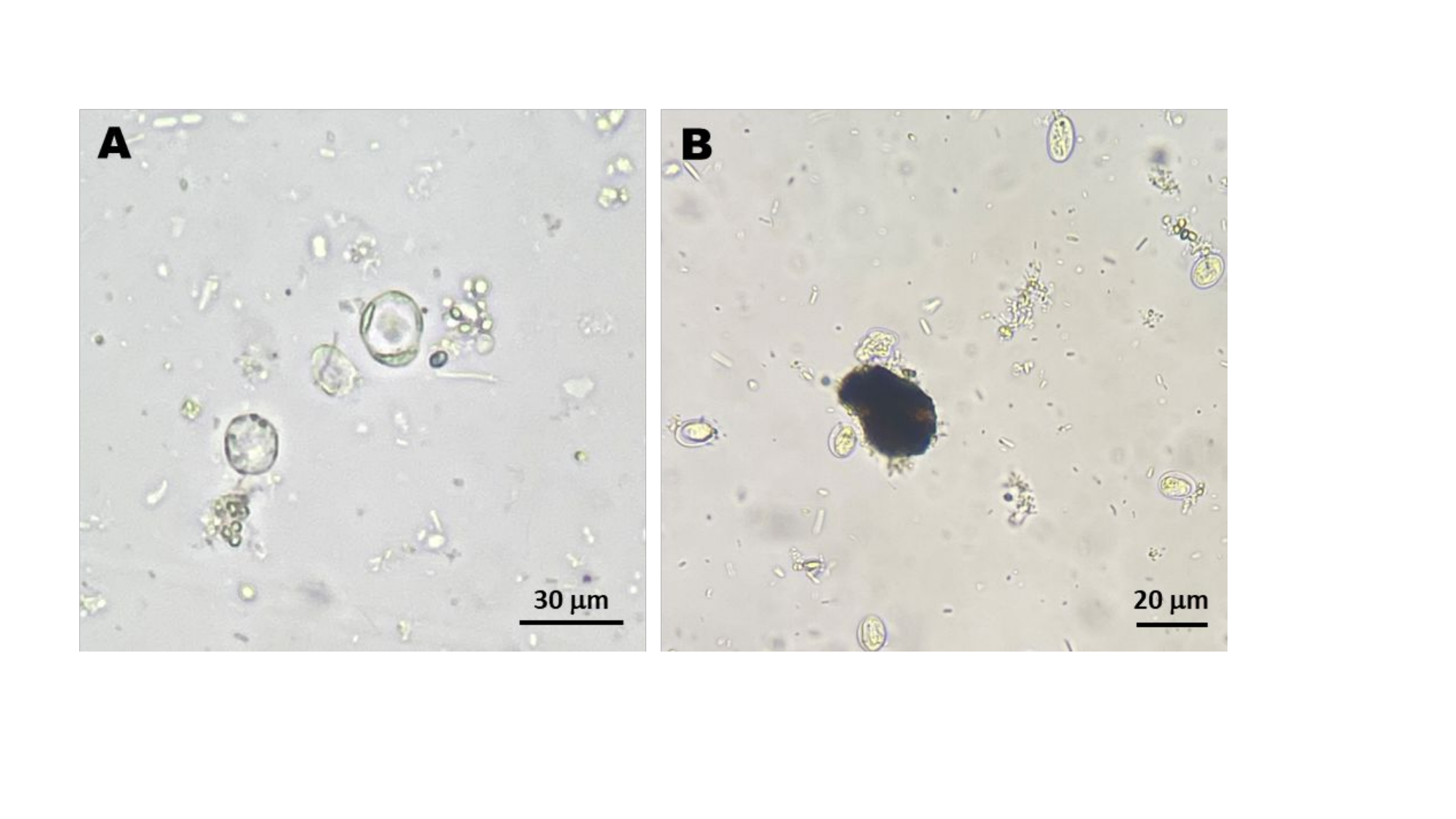

Supplement: Supplementary file 1 — Supplementary material 1: Microscopic images of Blastocystis (A) and Giardia duodenalis cysts (B) (40X magnification) [file mmc1.pptx]

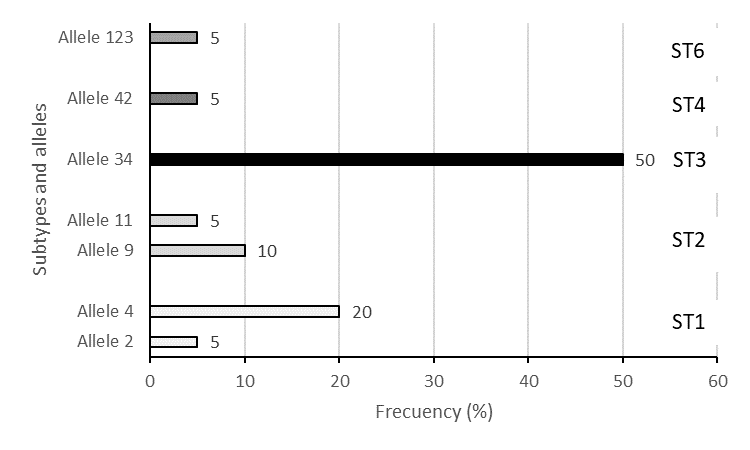


Supplemental Figure 3

Supplement: Supplementary file 3 — Supplementary material 3: Allele analysis (%) of each Blastocystis subtype detected [file mmc3.docx]

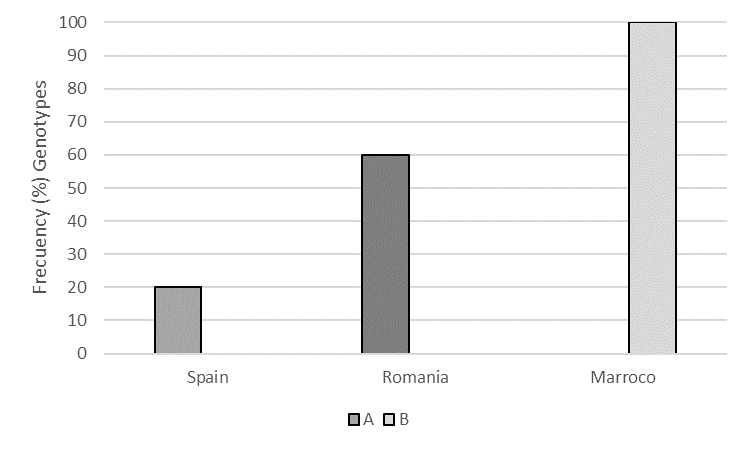


Supplemental Figure 4

Supplement: Supplementary file 4 — Supplementary material 4: Distribution of G. duodenalis assemblage A and B according to the inmate´s nationalityFootnoteView Edit Log13 [file mmc4.docx]
